# Advancing Gait Analysis: Integrating Multimodal Neuroimaging and Extended Reality Technologies

**DOI:** 10.3390/bioengineering12030313

**Published:** 2025-03-19

**Authors:** Vera Gramigna, Arrigo Palumbo, Giovanni Perri

**Affiliations:** 1 Department of Medical and Surgical Sciences, University “Magna Graecia”, Viale Europa, 88100 Catanzaro, Italy; info@arrigopalumbo.com; 2 Radiological Center Perri, 87100 Cosenza, Italy; giovanniperri@radiologiaperri.it

**Keywords:** gait analysis, multimodal methodologies, biomedical applications, extended reality

## Abstract

The analysis of human gait is a cornerstone in diagnosing and monitoring a variety of neuromuscular and orthopedic conditions. Recent technological advancements have paved the way for innovative methodologies that combine multimodal neuroimaging and eXtended Reality (XR) technologies to enhance the precision and applicability of gait analysis. This review explores the state-of-the-art solutions of an advanced gait analysis approach, a multidisciplinary concept that integrates neuroimaging, extended reality technologies, and sensor-based methods to study human locomotion. Several wearable neuroimaging modalities such as functional near-infrared spectroscopy (fNIRS) and electroencephalography (EEG), commonly used to monitor and analyze brain activity during walking and to explore the neural mechanisms underlying motor control, balance, and gait adaptation, were considered. XR technologies, including virtual, augmented, and mixed reality, enable the creation of immersive environments for gait analysis, real-time simulation, and movement visualization, facilitating a comprehensive assessment of locomotion and its neural and biomechanical dynamics. This advanced gait analysis approach enhances the understanding of gait by examining both cerebral and biomechanical aspects, offering insights into brain–musculoskeletal coordination. We highlight its potential to provide real-time, high-resolution data and immersive visualization, facilitating improved clinical decision-making and rehabilitation strategies. Additionally, we address the challenges of integrating these technologies, such as data fusion, computational demands, and scalability. The review concludes by proposing future research directions that leverage artificial intelligence to further optimize multimodal imaging and XR applications in gait analysis, ultimately driving their translation from laboratory settings to clinical practice. This synthesis underscores the transformative potential of these approaches for personalized medicine and patient outcomes.

## 1. Introduction

Human gait, a complex biomechanical and neurological process, is critical for mobility and serves as a key indicator of the overall health status. Abnormalities in gait patterns, such as alterations in the stride length, gait speed, cadence, and variability, can indicate a range of conditions, including neurological disorders (e.g., Parkinson’s disease, stroke), musculoskeletal injuries, and age-related functional decline.

These gait markers, particularly a reduced gait speed and stride length, are closely correlated with the functional status and independent living because they reflect underlying changes in an individual’s physical capacity and mobility. A slower gait speed is often indicative of weakened muscle strength, impaired coordination, or neurological dysfunction, all of which can limit a person’s ability to perform daily activities efficiently [1]. Similarly, a reduced stride length typically signals compromised mobility, often resulting from musculoskeletal pain, joint stiffness, or neurodegenerative diseases that affect movement control. Both markers are strongly linked to an increased risk of falls, as slower and shorter steps make it more challenging to maintain balance and respond to environmental hazards. These gait abnormalities can also lead to decreased independence, as individuals with impaired gait are more likely to require assistance with walking, performing basic tasks, or navigating their environment. Consequently, these gait markers serve as critical predictors of functional decline, providing valuable insights into a person’s overall health and quality of life and highlighting the need for timely intervention to maintain mobility and prevent further deterioration. Recent developments in gait analysis have primarily centered on either biomechanical data collection through motion capture or sensor technologies or on neuroimaging techniques such as fNIRS and EEG. However, the traditional approaches—such as observational assessments and conventional motion-tracking systems—are often limited in their ability to detect subtle neuromuscular variations, reducing their effectiveness in clinical applications [2]. In addition, these conventional methods often fail to provide a holistic understanding of gait, especially when considering both neural and biomechanical factors together.

As a result, precise gait analysis plays a fundamental role in clinical diagnosis, rehabilitation planning, and performance monitoring.

To address these limitations, modern gait analysis tools are necessary to identify these markers accurately and provide more detailed insights into the patient’s condition.

Recent advancements in eXtended Reality (XR) technologies [3,4,5,6] integrated with multimodal neuroimaging have revolutionized the field of gait analysis [7], providing novel opportunities to study both the biomechanical and neurological underpinnings of movement [8]. Multimodal neuroimaging techniques, including functional magnetic resonance imaging (fMRI), electroencephalography (EEG), and near-infrared spectroscopy (NIRS), offer insights into brain activity and neural pathways during gait, complementing data from motion capture systems, wearable sensors, and medical imaging modalities [9]. When combined with XR technologies—which cover virtual reality (VR), augmented reality (AR), and mixed reality (MR)—these tools create immersive, interactive environments that allow for real-time visualization, analysis, and feedback, enhancing therapeutic interventions and research capabilities. The main contribution of our review is to provide an in-depth exploration of the cutting-edge advancements in the field of advanced gait analysis, a multidisciplinary approach that combines the latest in neuroimaging techniques, extended reality technologies, and sensor-based methodologies to gain a deeper understanding of human locomotion. It highlights the role of wearable neuroimaging tools, such as fNIRS and EEG, which have become essential in monitoring and analyzing brain activity during walking. These technologies allow to investigate the neural mechanisms responsible for motor control, balance, gait adaptation, and the complex interactions between the brain and the musculoskeletal system. Additionally, the integration of XR technologies has revolutionized gait analysis by enabling the creation of immersive, interactive environments that simulate real-world scenarios. These XR technologies facilitate real-time gait simulation, movement visualization, and dynamic feedback, significantly enhancing the assessment of both the neural and biomechanical aspects of locomotion.

A state-of-the-art analysis [10] reviewed the integration of motion capture with functional neuroimaging techniques like EEG and fNIRS, aiming to enhance motor rehabilitation. The fusion of neurophysiological and behavioral data can provide new insights into cortical mechanisms during movement and improve rehabilitation practices. The review [10] highlighted that while EEG and motion capture were commonly used in the studies examined, synchronization techniques for the multimodal systems were underreported. It also noted that the combination of these features helped identify movement-related cortical activity, but statistical methods for analyzing cortico-kinematic relationships were not widely utilized. The conclusion stressed that while this fusion holds promise for improving motor rehabilitation, further research is needed to advance synchronization, validate multimodal parameters, and improve the usability of these technologies in clinical environments.

In comparison, our review emphasizes the role of extended reality technologies in gait analysis and their integration with neuroimaging tools like EEG, and fNIRS, along with sensor-based methodologies. Our focus is on understanding both the biomechanical and neurological components of gait, with an emphasis on creating immersive, real-time feedback environments. In addition, our review highlights the innovative potential of XR technologies for real-time gait simulation and dynamic feedback, something that is not emphasized in previous studies. We examine the applications of advanced gait analysis in clinical and research settings, outline technical and practical challenges, and propose future directions for the field. By synthesizing the current advances, our work aims to provide a roadmap for leveraging these innovative tools to improve gait analysis, ultimately driving the advancement of personalized healthcare and enhancing patient outcomes.

## 2. Research Methodology

### 2.1. Search Strategy

This systematic review adhered to the Preferred Reporting Items for Systematic Reviews and Meta-Analyses (PRISMA) guidelines [11]. A comprehensive literature search was conducted on 14 December 2024, utilizing major engineering and medical databases, including IEEE Xplore, PubMed, ScienceDirect, and Scopus, as outlined in Table 1. The review focused on English-language publications from 2018 to 2024 with available abstracts. To ensure relevance to the scope of the study, specific keywords were carefully defined and applied during the search process. The following structured search string was used to structure this paper: “neuroimaging” AND—“Extended Reality” OR “Augmented Reality” OR “Virtual Reality” OR “Mixed Reality”—AND—“Gait Analysis”. In addition, articles identified through the reference lists of previously retrieved articles were included to increase the likelihood that all the relevant studies were identified.

### 2.2. Inclusion and Exclusion Criteria

Articles were included in the review if they met the following criteria: (1) they utilized at least one imaging methodology and one extended (virtual, augmented, or mixed) reality approach in a gait analysis context; (2) demonstrated the feasibility, effectiveness, or applicability of multimodal imaging and extended reality methodologies within a gait analysis context, either partially or fully; (3) dealt with the analysis of real and not virtual or simulated walking (balance control tests were also considered); (4) presented complete research; and (5) were written in English.

The exclusion criteria included the following: (1) studies where a combination of imaging and extended reality technologies was not adopted or was not clearly specified; (2) research involving multimodal approaches in animal studies rather than human contexts; (3) applications outside the gait analysis domain; and (4) articles without an available full text. Additionally, books, book chapters, letters, review articles, editorials, and short communications were excluded from consideration.

### 2.3. Study Selection

**Figure 1:**
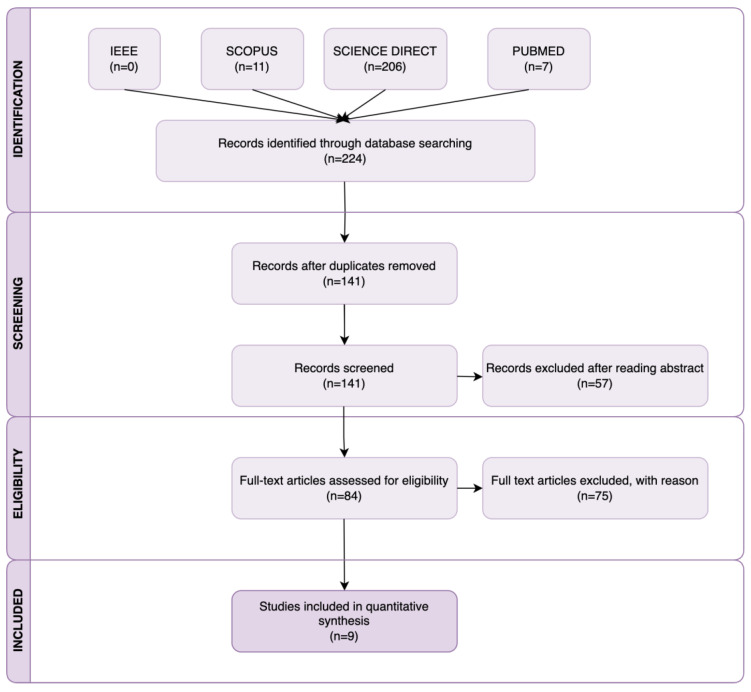
Flow chart of search strategy and study selection according to PRISMA guidelines.

Through carefully searching the databases and additional sources, a total of 224 search results were identified. After removing all duplicates, 141 studies underwent title and abstract screening, and the inclusion criteria were examined. The full texts of 84 papers assessed for eligibility were carefully analyzed. A total of 69 articles [12,13,14,15,16,17,18,19,20,21,22,23,24,25,26,27,28,29,30,31,32,33,34,35,36,37,38,39,40,41,42,43,44,45,46,47,48,49,50,51,52,53,54,55,56,57,58,59,60,61,62,63,64,65,66,67,68,69,70,71,72,73,74,75,76,77,78,79] were excluded due to exclusion criterion (1), 1 contribution [80] due to the exclusion criterion (2) and 5 scientific results [81,82,83,84,85] due to exclusion criterion (3). Finally, only 9 studies were included in the quantitative synthesis. Figure 1 illustrates the methodological approach used. To support analysis and comparisons, all existing integrated neuroimaging and XR technology solutions and the associated system parameters are comprehensively summarized and discussed in Section 4.

## 3. Advanced Gait Analysis Approach and Key Components

**Figure 2:**
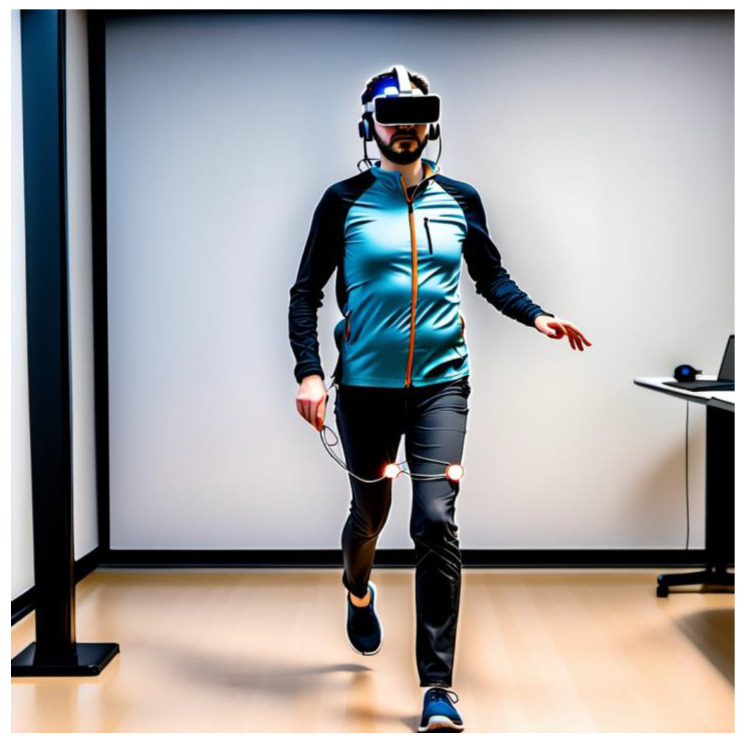
Example of advanced gait analysis system and key components.

**Figure 3:**
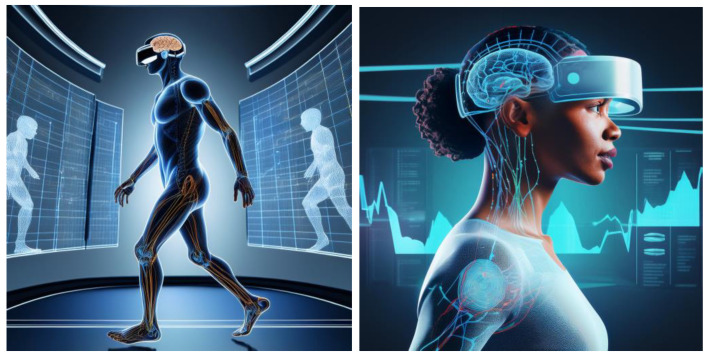
Examples of advanced gait analysis system and key components.

Advanced gait analysis (Figure 2 and Figure 3) can be defined as a multidisciplinary approach aiming to study human locomotion by integrating neuroimaging techniques, extended reality technologies, and sensor-based methodologies to evaluate walking performance. This innovative framework seeks to enhance the understanding of gait by exploring both its cerebral and biomechanical aspects, offering deeper insights into how the brain and musculoskeletal system coordinate and control movement. In addition, the incorporation of extended reality technologies, such as virtual, augmented, and mixed reality, through head-mounted displays allow for immersive and interactive environments for gait analysis, the simulation of real-world scenarios, and the visualization of movement in real time, enabling a more comprehensive assessment of locomotion and its interplay with neural and biomechanical factors. The key components of this approach are as follows. 

### 3.1. Neuroimaging Techniques

Several neuroimaging modalities, such as functional near-infrared spectroscopy (fNIRS), electroencephalography, and functional magnetic resonance imaging (fMRI), are commonly used to monitor and analyze brain activity during walking. The role of these tools is to explore the neural mechanisms underlying motor control, balance, and gait adaptation. Considering the aim of this review, we focalized our attention only on wearable neuroimaging modalities (EEG and fNIRS) suitable for the analysis of real and not virtual or simulated walking.

### 3.2. Extended Reality (XR) Technologies

Extended reality technologies include virtual reality, augmented reality, and mixed reality, typically facilitated through head-mounted displays (HMDs). XR environments enable the creation of controlled, immersive scenarios that simulate real-world challenges, offering real-time feedback and visualizations for both assessment and rehabilitation.

### 3.3. Sensor-Based Systems

Wearable sensors in gait analysis are compact and portable devices designed to capture biomechanical data during locomotion. These sensors, including accelerometers, gyroscopes, magnetometers, and inertial measurement units (IMUs), measure the spatio-temporal, kinetic, and kinematic parameters of human gait. By enabling the quantification of gait dynamics outside traditional lab settings, wearable sensors offer new opportunities for personalized diagnostics and treatment strategies. Additionally, portable electromyography (EMG) sensors play a crucial role in advanced gait analysis by providing real-time data on muscle activity during locomotion. These sensors allow for the assessment of muscle function and coordination, offering valuable insights into the neuromuscular control of gait, which complements the biomechanical data captured by other wearable sensors.

## 4. Advanced Gait Analysis: Literature Results

Regarding the literature results, only nine existing applications of gait analysis based on the integration of multimodal neuroimaging, extended reality technologies, and sensor-based systems are included and illustrated. These studies are carefully analyzed and their main characteristics in terms of brain imaging acquisition, extended reality technology typologies, gait analysis performance evaluation, and the number and type of participants are summarized in Table 2. The studies are tabulated in chronological order.

The studies included various wearable imaging technologies such as EEG, fNIRS, motion capture, and sensor-based systems and augmented and virtual reality solutions. Below, we summarize the key findings, highlighting both qualitative and quantitative results.

The study by Zhang et al. [86] investigated the effectiveness of combining low-frequency repetitive transcranial magnetic stimulation (rTMS) with gait-adaptive training to improve lower limb function and brain regulatory mechanisms in subacute stroke recovery. It involved 27 patients with subacute hemiparesis, divided into experimental and control groups. Both groups participated in gait adaptability training five times a week for 4 weeks, with the experimental group receiving daily rTMS before each session.

The results showed that both groups experienced significant improvements in motor function (measured by the Fugl-Meyer Assessment, a 10 m walk test, and the Berg Balance Scale) after the 4-week intervention. However, the experimental group showed greater improvements in the Fugl-Meyer Assessment (*p* = 0.024) and 10 m walk test (*p* = 0.033). Additionally, the experimental group had a more significant decrease in the brain symmetry index, a marker of brain function (*p* = 0.026). The cortical subgroup within the experimental group had significantly lower brain symmetry index values compared to the control group (*p* = 0.006). In conclusion, combining low-frequency rTMS with gait-adaptive training enhanced lower limb function and brain regulatory mechanisms more effectively than gait adaptation training alone, suggesting a promising approach for subacute stroke rehabilitation.

A mixed-methods feasibility study by Gomaa [87] aimed to develop a new assessment method for evaluating the fear of falling (FOF) during motion and walking in virtual environments, specifically focusing on people with Parkinson’s disease (PD). The study addressed various FOF-related factors, including cognitive responses, neuromuscular reactions, and postural stability. It involved four phases: the first two phases assessed individuals without PD, and the last two included participants diagnosed with PD. Data were collected from questionnaires, neurophysiological assessments, the measurement of movement and gait parameters, and usability and acceptability evaluations. Additionally, semi-structured interviews were conducted with both participants and research assistants to gather information on their experiences during the assessments. Descriptive statistics were used to report demographic data, assessment scores, and the feasibility, usability, and acceptability of the methods. Movement and gait outcomes, as well as neurophysiological data, were extracted and analyzed. The relationships between different factors were examined using a regression model. Thematic analysis was applied to manage and analyze the qualitative data gathered from the interviews.

Maas and colleagues [88] presented a proof-of-concept study with the aim to create and validate a system capable of simultaneously collecting and synchronizing, in real time, data on brain activity (using mobile EEG and fNIRS), kinetic, and kinematic gait parameters in an immersive virtual reality environment. This integrated setup aimed to facilitate foundational research into the neural correlates of gait during real-time walking. Specifically, the study focused on two objectives: first, verifying synchronization among gait measurements, including those taken by force plates, motion capture systems, and electromyography; and second, ensuring synchronization between neurophysiological systems (EEG and fNIRS) and the gait measurements. This study highlighted the attainable temporal precision in synchronizing neurophysiological, kinematic, and kinetic data collection within the Gait Real-Time Analysis Interactive Lab system (GRAIL; Motekforce Link, Amsterdam, the Netherlands).

The scientific contribution of Daşdemir et al. [89] explored various locomotion techniques, presenting a dataset named VREEG (https://eegdatasets.erzurum.edu.tr/Home/VREEG, accessed on 7 January 2025) collected for this purpose. This study focused on predicting the physical and psychological effects of virtual reality locomotion, such as cybersickness (CS), eyestrain, disorientation, and psychological issues like addiction or detachment from reality. Thirty-two participants were involved, completing ten different locomotion techniques in VR. The study utilized both objective and subjective measures to assess physiological responses, usability, and cybersickness. A predictive model was developed using EEG features extracted from the time, frequency, and time–frequency domains. This model achieved 99% accuracy in predicting nausea, oculomotor issues, and disorientation levels among the participants. The study also highlighted the effectiveness of teleportation techniques (such as shift and blink locomotion), which resulted in fast transitions and lower levels of cybersickness. These findings are significant for understanding the potential risks of VR locomotion and offer insights into how to mitigate these risks while optimizing the benefits of VR technology.

The group of Stojan et al. [90] assessed brain activity in the prefrontal cortex (PFC) and parietal lobe (PL) to investigate whether higher PFC activation during a virtual reality dual-task (DT) walking test in older adults was related to compensation, dedifferentiation, or neural inefficiency. 

More specifically, fifty-six healthy older adults participated in the study, completing three tasks: treadmill walking (1 m/s), a Stroop task, and a Serial 3 s task, under single-task (ST) and DT conditions (walking + Stroop, walking + Serial 3 s). The DT walking test was performed with the Gait Real-Time Analysis Interactive Lab. Gait performance was assessed via ground reaction forces. In addition, the study measured brain activity using functional near-infrared spectroscopy over the ventrolateral and dorsolateral PFC (vlPFC, dlPFC) and inferior and superior PL (iPL, sPL), as well as behavioral outcomes like the step time variability (walking), Balance Integration Score (Stroop), and number of correct calculations (S3_corr_ (Serial 3 s). The neurophysiological outcomes were the oxygenated (HbO) and deoxygenated hemoglobin (HbR).

The quantitative results showed an expected upregulation in brain activity from the ST to DT conditions, with the increase being more pronounced in PFC (especially the vlPFC) than in PL regions. The changes in brain activation were positively correlated across all brain regions. Furthermore, higher brain activation changes during DT walking were associated with greater declines in behavioral performance (e.g., slower walking, more errors in cognitive tasks). These findings suggest that the increased brain activation observed during DT walking in older adults is likely due to neural inefficiency and dedifferentiation, rather than compensation.

In another study [91], the authors evaluated postural control performance under various visual conditions using a virtual reality system (HTC Vive Pro, HTC America Inc., Seattle, WA, USA) while simultaneously monitoring cortical activity through a functional near-infrared spectroscopy system. A total of 24 healthy participants were recruited. Postural stability and cortical responses to perturbations were assessed under multiple scenarios involving both postural and visual perturbations. Under all conditions, the participants’ COP position was recorded using a tactile sensor sheet (BIG-MAT™; Nitta Corporation, Osaka, Japan) and the electromyography data recording was performed by using a wireless surface EMG system (WEB-1000; NIHON KOHDEN Corporation, Tokyo, Japan). The postural stability results in response to visual and postural perturbations demonstrated that, under all conditions, the COP sway and EMG activity ratio were significantly greater than one, indicating that postural reactions were triggered in all conditions. Visual perturbations without postural perturbations (VISUAL condition) did not lead to statistically significant cortical activation in this study. In contrast, postural perturbations caused a significant increase in the oxy-Hb signal, irrespective of the visual stimulus condition. The findings revealed that the combination of visual and postural perturbations significantly enhanced cortical activity in the supplementary motor area and the superior parietal lobe.

In the study by Piazza et al. [92], the authors aimed to develop and evaluate a system for multimodal gait analysis during virtual reality-based gait training in pediatric populations. The analysis focused on EEG correlates as well as the kinematic and kinetic parameters of gait. To achieve this, an EEG system was successfully integrated with the Gait Real-Time Analysis Interactive Lab (GRAIL). During the session, gait kinematics and kinetics and EMG data were also acquired. The results in terms of the triggering performance showed that the Phidget system that synchronized EEG signals with kinematic and kinetic data achieved high sensitivity (96.55%) and a high critical success index (CSI) (96.29%) in detecting the initial contact (IC) during gait. The sensitivity and CSI were higher in healthy adults (96.68% sensitivity, 98.39% CSI) than in children (90.97% sensitivity, 90.95% CSI), particularly because many children had cerebral palsy (CP), which affects gait reliability.

As the Phidget triggers could not distinguish between right and left steps, gastrocnemii EMG signals were simultaneously recorded to enable this identification, and these were also suggested as an alternative off-line trigger signal for step recognition. The EMG signal showed an average sensitivity of 99.09% and a CSI of 98.92% for healthy adults, with lower but still good values for children (92.68% sensitivity, 91.65% CSI). A combined trigger system integrating both Phidget and EMG signals improved performance, with a sensitivity of 99.96% and CSI of 99.63% for adults and a sensitivity of 99.39% and CSI of 99.26% for children. This combined system provided more reliable synchronization, recovering missing information from either trigger alone and ensuring higher reliability in analyzing pathological gait patterns.

The purpose of the study by the Peterson group [93] was to quantify differences in cortical and muscular connectivity patterns in response to brief sensorimotor perturbations that challenged postural control during walking and standing. For balance perturbations, a 20-degree field-of-view rotation or a pull at the waist were used. The field-of-view rotation was performed using a virtual reality headset (Oculus Rift DK2, Oculus, Redmond, WA, USA). A 136-channel EEG system (BioSemi Active II, BioSemi, Amsterdam, the Netherlands) and eight lower leg electromyography channels (Vicon, Los Angeles, CA, USA) were also used. The results indicated reduced connectivity between the occipito-parietal regions during visual rotations, along with widespread increases in connectivity in central areas during pull perturbations. Additionally, evidence of communication between the cortex and muscles during perturbed balance was observed. These findings demonstrate that sensorimotor perturbations to balance modify cortical networks and can be measured through effective connectivity estimation.

Another study [94] aimed to utilize functional near-infrared spectroscopy to assess whether individuals with visual vertigo (VV) exhibit distinct cerebral activation patterns during optic flow compared to control subjects. Fifteen individuals with visual vertigo, along with fifteen healthy controls (CON), stood in a virtual reality environment (modified NeuroTest platform, NeuroCom International, Inc., Clackamas, OR, USA) that recorded ground reaction forces and viewed an anterior–posterior optic flow. The support surface was either fixed or sway-referenced. The evaluated forces were then used to calculate the center of pressure. An electromagnetic tracking system (Polhemus Fastrak, Colchester, VT, USA) was employed to measure the postural sway. Changes in cerebral activation were recorded using fNIRS during periods of optic flow relative to a stationary visual environment. The main results of this study confirmed that, compared with the CON group, the VV group displayed decreased activation in the bilateral middle frontal regions when viewing optic flow while standing on a fixed platform. Postural sway was greater in the VV group than in the CON group. While preliminary, these findings contribute to the expanding body of literature employing functional brain imaging to investigate cerebral activation changes in individuals reporting dizziness, disorientation, and unsteadiness.

**Figure 4:**
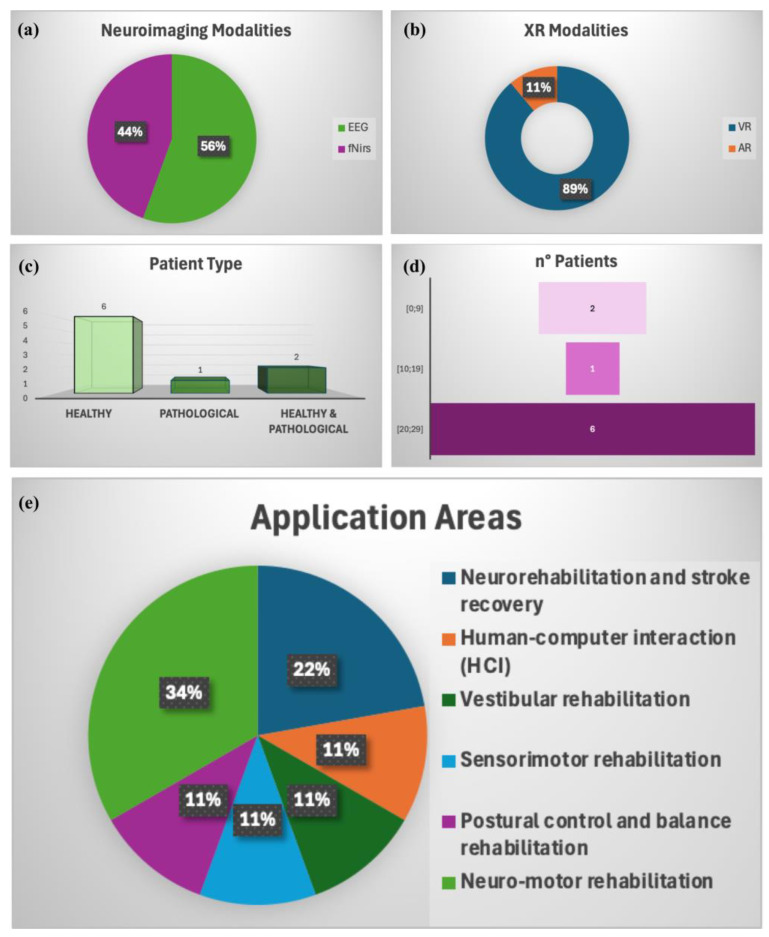
Distribution of results in terms of (**a**) neuroimaging modalities, (**b**) XR modalities, (**c**) patient types (healthy vs. pathological groups), (**d**) number of participants in class bins (e.g., 0–9, 10–19, 20–29, etc.), and (**e**) main applications.

An overall and immediate picture of the results of our review in terms of the existing solutions is provided in Figure 4. More specifically, it quantifies the distribution of the nine studies in terms of (a) neuroimaging modalities, (b) XR modalities, (c) patient types (healthy vs. pathological groups), (d) the number of participants in the class bins (e.g., 0–9, 10–19, 20–29, etc.), and (e) the main applications.

## 5. Discussion

Recent advances in gait analysis have typically focused on either biomechanical data collection through motion capture or sensor technologies or on neuroimaging modalities like fNIRS and EEG. However, these traditional methods often lack a comprehensive understanding of gait, particularly when examining neural and biomechanical factors in tandem.

The integration of multimodal neuroimaging and extended reality technologies represents a significant advancement in the field of gait analysis. By combining detailed insights into neural activity with immersive, interactive environments, this approach overcomes several limitations of conventional gait analysis methods.

One of the most notable contributions of advanced gait analysis is the integration of neuroimaging techniques (such as EEG and fNIRS) with extended reality technologies and sensor-based systems to enhance our understanding of neural and biomechanical interactions and thus to elucidate the complex interplay between neurological processes and biomechanical movements during gait. These technologies enable the observation of brain activity in real time, offering insights into neural plasticity, motor control, and coordination deficits associated with conditions such as Parkinson’s disease, stroke, and age-related decline. When paired with XR platforms, researchers and clinicians can visualize these interactions dynamically, allowing for targeted therapeutic interventions and personalized rehabilitation plans.

The use of XR technologies, encompassing virtual, augmented, and mixed reality, has demonstrated their ability to create realistic and controlled environments that simulate daily walking scenarios. This capability not only facilitates detailed biomechanical analyses but also enables real-time feedback and adaptive training protocols. For instance, virtual environments can be tailored to challenge specific aspects of gait, such as balance, the stride length, or cadence, while simultaneously monitoring neural responses. Such immersive experiences have been shown to enhance patient engagement, motivation, and compliance, leading to improved therapeutic outcomes.

Our review emphasizes the integration of neuroimaging techniques like functional near-infrared spectroscopy and EEG with biomechanical and extended reality technologies, offering a more holistic approach to understanding human locomotion. While previous studies have tended to examine brain activity or movement mechanics independently [95,96], the combination of these modalities described in our review enhances both the accuracy and depth of gait analysis by investigating motor control, balance, and gait adaptation from a multidimensional perspective.

Moreover, our review stands out by incorporating XR technologies (virtual, augmented, and mixed reality), which provide immersive environments for gait analysis. This represents a significant leap forward from conventional methods like motion capture and video analysis, which often lack the interactive and adaptive qualities offered by XR. XR allows for the simulation of dynamic, real-world environments, providing clinically relevant and ecologically valid assessments. This ability to create realistic environments also enables real-time simulations and detailed movement visualizations, offering unique advantages for rehabilitation and clinical decision-making.

The findings from our systematic review provide evidence for the feasibility, effectiveness, and applicability of these innovative tools, offering a transformative pathway for clinical and research applications in gait analysis.

Feasibility: Several studies demonstrated the successful integration of advanced technologies like EEG systems, functional near-infrared spectroscopy, and motion capture systems with virtual reality environments to measure brain activity, gait kinematics, and kinetics. Specifically, the study by Maas and colleagues [88] showcased the real-time synchronization of EEG and fNIRS data with gait measurements, providing a solid foundation for future research on the neural correlates of gait during walking. Moreover, the multimodal system developed in the work by Piazza et al. [92] achieved high sensitivity (96.55%) and critical success index (CSI) values for step detection, demonstrating the feasibility of combining EEG with motion-tracking technology for accurate gait analysis.

Effectiveness: The studies reviewed highlight the effectiveness of these systems in improving gait performance and enhancing our understanding of brain function during walking. For example, the study by Zhang et al. [86] reported greater improvements in motor function and the brain symmetry index in the experimental group using low-frequency rTMS combined with gait-adaptive training, indicating the potential benefits of integrating neurostimulation with gait training. Similarly, the work of Stojan et al. [90] revealed that dual-task walking tasks in older adults led to significant brain activation in the prefrontal and parietal regions, which could be quantified using fNIRS technology. These findings underscore the effectiveness of advanced systems for measuring and analyzing neural and behavioral responses to gait training.

Applicability: The systems reviewed in this study offer promising applications for clinical and research settings. In particular, the combined Phidget and EMG trigger system, as reported by Piazza et al. [92], improved performance by synchronizing neural and kinematic data, thus making it easier to analyze pathological gait patterns and enhance rehabilitation outcomes. Additionally, the use of VR environments in several studies (such as those by Gomaa [87] and Stojan [90]) allowed for assessing balance and gait under various perturbation conditions, providing valuable insights for developing targeted rehabilitation programs for patients with conditions like Parkinson’s disease or stroke.

The visual representation of the results of our review quantifies the distribution of the nine included studies in terms of the neuroimaging modalities, XR modalities, patient types (healthy vs. pathological groups), number of participants in the class bins (e.g., 0–9, 10–19, 20–29, etc.), and main applications.

Regarding neuroimaging methods, the results of this review demonstrate that EEG and fNIRS are the most widely used thanks to their ability to evaluate, through wearable systems, brain activity during the execution of complex motor tasks. EEG excels in assessing electrical brain activity, while fNIRS evaluates hemodynamic changes. Indeed, EEG is highly useful for capturing the real-time dynamics of brain activity, especially for investigating cognitive processes, motor control, and adjustments during walking. It provides fast, dynamic data but lacks the precise localization of brain activity. fNIRS provides more localized information regarding brain areas involved in gait, especially useful for monitoring blood oxygenation changes in specific cortical regions like the motor cortex. It is slower than EEG but offers better spatial precision and can still be used effectively in real-world walking scenarios. In an advanced gait analysis setup, these two techniques could be integrated to provide a comprehensive view of both the neural and biomechanical aspects of human locomotion.

Concerning XR technologies, the studies included in this review highlight the fact that only one study [86] used augmented reality, while the others were focused on virtual reality. Therefore, the advantages of this technology are not fully known in the field of gait analysis. Furthermore, motor performance has commonly been assessed by means of electromyographic sensors or force platforms. This highlights the need for further studies using wearable inertial sensors that provide the possibility of extracting numerous kinetic, kinematic, and spatio-temporal parameters in real time.

The nine analyzed studies mainly concentrated on a cohort of controls, and the most frequent number of subjects was in the (20–29) class.

In addition, the studies included in this review highlighted diverse applications of these combined methodologies:

- Comprehensive Analysis: By merging brain activity measurements with physical movement data, researchers can better understand how neurological and mechanical factors influence walking.
- Rehabilitation: XR environments allow for innovative therapeutic interventions, such as retraining gait patterns in individuals with neurological disorders or injuries.
- Personalized Medicine: These approaches enable tailored interventions based on individual gait patterns and neural responses.
- Scientific Exploration: These approaches advance the study of locomotion under various physiological and environmental conditions.

The approach described in our review represents a significant leap forward in studying human gait, integrating cutting-edge technologies to enhance clinical diagnostics, therapeutic strategies, and scientific research in mobility and motor control. In clinical settings, the integration of multimodal imaging and XR has proven effective in diagnosing subtle gait abnormalities that may not be detectable through traditional observational assessments. Moreover, these tools offer robust frameworks for evaluating the efficacy of interventions, enabling clinicians to make data-driven decisions. In research contexts, this approach provides unparalleled opportunities to explore the underlying mechanisms of gait, advancing our understanding of neuromuscular and neuroplastic responses to various conditions and treatments.

Although in recent years there has been an attempt to adopt an advanced gait analysis approach by integrating imaging techniques and extended reality and sensor-based technology, this line of research needs further investigation.

Despite the promising potential of these technologies, the current approaches to gait analysis integrating multimodal neuroimaging, wearable sensors, and XR technologies face several limitations. Indeed, several technical and practical challenges must be addressed to ensure their widespread adoption.

The primary challenges include the technical complexity and high cost of these systems, the need for specialized training to operate them, and issues with synchronizing multimodal data. Furthermore, variability in study designs, data acquisition protocols, and analysis methods across the reviewed studies underscores the need for standardized frameworks to ensure the reproducibility and comparability of findings. The lack of standardized protocols for data acquisition and outcome measurement across studies makes it difficult to compare findings and establish uniform conclusions. While some studies have demonstrated short-term improvements, there is a need for more research to assess the long-term effectiveness and real-world applicability of these technologies.

Further investigation is needed to develop standardized frameworks for data acquisition, system integration, and outcome evaluation, which would ensure more consistent and reproducible results. Long-term studies are essential to understand the sustained benefits of using XR technologies combined with neuroimaging for gait rehabilitation, particularly in chronic conditions. Expanding research to include larger and more diverse participant groups, including elderly individuals and children, will help assess the broad applicability of these approaches. Additionally, technological advancements are necessary to improve the realism of XR environments, reduce side effects like motion sickness, and enhance user comfort. Investigating the underlying neural mechanisms involved in gait, particularly how virtual environments influence neuroplasticity, is another key area for future exploration. Finally, making these systems more affordable and user-friendly will be crucial for their widespread adoption in clinical and home settings.

## 6. Conclusions

The integration of multimodal neuroimaging and XR technologies represents a paradigm shift in gait analysis, offering unprecedented insights into the neural and biomechanical aspects of human movement. By addressing current limitations and leveraging emerging technologies, this approach has the potential to transform clinical diagnosis, rehabilitation, and research, ultimately improving patient outcomes and advancing the field of personalized healthcare. To fully realize the potential of multimodal neuroimaging and XR in gait analysis, future research should focus on developing cost-effective, user-friendly systems that can be seamlessly integrated into routine clinical practice. Advances in machine learning and artificial intelligence may further enhance data processing and interpretation, enabling real-time decision-making and personalized interventions. Additionally, longitudinal studies with larger sample sizes are needed to establish the long-term benefits and reliability of these approaches. Collaborative efforts between researchers, clinicians, and technology developers will be essential to address existing challenges and drive innovation in this rapidly evolving field.

## Figures and Tables

**Table 1:** Databases used for this review.

Database Name	URL	Date Accessed
IEEEXplore	https://ieeexplore.ieee.org/Xplore/home.jsp	14 December 2024
Science Direct	https://www.sciencedirect.com/	14 December 2024
Scopus	https://www.scopus.com/	14 December 2024
Pubmed	https://pubmed.ncbi.nlm.nih.gov/	14 December 2024

**Table 2:** Summary of existing advanced gait analysis applications. Main characteristics in terms of brain imaging acquisition, extended reality technology typologies, gait analysis performance evaluation, and number and type of participants.

Reference	Year	Aim	Methodology	Participants (*n*, Type)
Zhang et al. [86]	2024	To explore the efficacy of combining rTMS with gait-adaptive training to enhance lower limb function and regulatory mechanisms in subacute stroke.	-Neuroimaging: EEG (actiCHamp) -Extended Reality: AR -Sensor-Based Systems: C-Mill smart gait training system (C-Mill, Motekforce Link BV)	27 patients with subacute hemiparesis (18–75 years)
Gomaa et al. [87]	2024	To develop an assessment method for FOF while in motion and walking within virtual environments.	-Neuroimaging: EEG (ANT Neuro, Hengelo, the Netherlands) -Extended Reality: VR (Meta Quest 3) -Sensor-Based Systems: Postural IMU; Trigno wireless EMG system (Delsys, Natick, MA, USA)	10 to 30 participants, people without PD and people with PD
Maas et al. [88]	2024	To develop and validate a setup that allows for the simultaneous collection and real-time synchronization of brain activity (via mobile EEG and fNIRS), kinetic, and kinematic gait measurements.	-Neuroimaging: fNIRS (two 8 × 8 NIRSport 2.0 systems (NIRx Medical Technologies, Glen Head, NY, USA); EEG (LiveAmp, Brain Products GmbH, Gilchingen, Germany) -Extended Reality: VR -Sensor-Based Systems: Marker-based, passive, optical motion detection system (VICON Motion Systems Ltd.; Oxford, UK), two ground reaction force plates (Motek Medical; Utrecht, the Netherlands), and an external EMG measuring system (Cometa; Bareggio, Italy)	3 volunteers (1 M, 2 F, 22–37 years)
Daşdemir et al. [89]	2023	To investigate changes in objective brain activity (EEG) and subjective simulatory sickness questionnaire (SSQ) scores according to an individual’s susceptibility to VR locomotion.	-Neuroimaging: EEG (Emotiv EPOC Flex) -Extended Reality: VR (HTC-Vive Lighthouses) -Sensor-Based Systems: n.d.	32 volunteers (21 M, 11 F, aged 18–30)
Stojan et al. [90]	2023	To assess brain activity in the PFC and parietal lobe and to investigate whether higher PFC activation during DT walking in older adults is related to compensation, dedifferentiation, or neural inefficiency.	-Neuroimaging: fNIRS (NIRSport systems, NIRx Medical Technologies, Glen Head, NY, USA) -Extended Reality: VR (D-Flow, Motekforce Link, Amsterdam, the Netherlands) -Sensor-Based Systems: Vicon Nexus (v2.10)	56 healthy older adults (30 F, aged 64 –79)
Nishimoto et al. [91]	2023	To investigate postural control performance under different visual conditions using a virtual reality system, simultaneously measuring cortical activities with a functional near-infrared spectroscopy system.	-Neuroimaging: 50-channel NIRS system (OMM 3000; Shimadzu Corporation, Kyoto, Japan) -Extended Reality: VR (HTC Vive Pro, HTC America Inc., Seattle, WA, USA) -Sensor-Based Systems: Wireless surface EMG (WEB-1000; NIHON KOHDEN Corporation, Tokyo, Japan)	24 healthy participants (11 M, 13 F, aged 19–42 years)
Piazza et al. [92]	2021	To set up and test a system for the multimodal analysis of the gait pattern during the VR gait training of pediatric populations by analyzing the EEG correlates as well as the kinematic and kinetic parameters of the gait.	-Neuroimaging: EEG system (eegoTMmylab (ANT Neuro, Hengelo, The Netherlands)) -Extended Reality: VR GRAIL (Motek Medical, Houten, The Netherlands) -Sensor-Based Systems: Vicon motion capture system (Oxford Metrics, Oxford, UK)	5 healthy adult volunteers (mean age = 30.9 years; 2 M) 4 children (mean age = 11.2 years; 1 M healthy child; 3 children with a diagnosis of unilateral CP, 2 M)
Peterson et al. [93]	2019	To quantify differences in group-level corticomuscular connectivity responses to sensorimotor perturbations during walking and standing.	-Neuroimaging: 136-channel EEG (BioSemi Active II, BioSemi, Amsterdam, NL) -Extended Reality: VR (Oculus Rift DK2, Oculus, Redmond, WA, USA) -Sensor-Based Systems: 8 lower leg EMG channels (Vicon, Los Angeles, CA, USA)	30 healthy young adults (15 F, 15 M, aged 22.5 ± 4.8 years)
Hoppes et al. [94]	2018	To determine if individuals with visual vertigo have different cerebral activation during optic flow compared with control subjects.	-Neuroimaging: fNIRS (CW6 real-time system; TechEn, Inc.; Milford, MA, USA) -Extended Reality: VR -Sensor-Based Systems: Ground reaction forces	15 healthy controls (5 M, 10 F, aged 18–65)

Associated acronyms: EEG: electroencephalography; EMG: electromyography; fNIRS: functional near-infrared spectroscopy; FOF: fear of falling; VR: virtual reality; AR: augmented reality; IMU: inertial measurement unit.

## Data Availability

No new data were created or analyzed in this study. Data sharing is not applicable to this article.
